# Unilateral diaphragmatic paralysis and complete brachial plexus injury in a complex birth injury: A rare case report mimicking Tarlov cyst and review of literature

**DOI:** 10.1002/ccr3.8406

**Published:** 2024-01-03

**Authors:** Khadijeh sadat Najib, Seyyed Mostajab Razavinejad, Fatemeh Yarmahmoodi, Hamide Barzegar

**Affiliations:** ^1^ Neonatal Research Center Shiraz University of Medical Sciences Shiraz Iran; ^2^ Assistant Professor, Neonatal Research Center Shiraz University of Medical Sciences Shiraz Iran; ^3^ Assistant Professor of Radiology, Medical Imaging Research Center Shiraz University of Medical Sciences Shiraz Iran; ^4^ Neonatal Research Center Shiraz University of Medical Sciences Shiraz Iran

**Keywords:** birth injury, newborn, perineural cyst, Tarlov cyst

## Abstract

The rare occurrence of Tarlov cysts in pediatric patients, particularly in the context of complex birth injuries, necessitates thorough evaluation and tailored management approaches. A comprehensive understanding of the clinical significance and optimal treatment strategies for this unique combination is crucial to ensure effective and individualized care for affected children.

## Graphical abstract

The occurrence of complex birth injury concurrent with Tarlov cyst is a rare condition. The clinical significance and management of this coexistence remain poorly understood in neonates, necessitating further comprehensive evaluations.

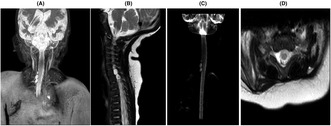

## 1 INTRODUCTION

Birth injuries during the birth process remain a concern within the field of neonatology. Although there has been a decline in the occurrence and mortality rates of such injuries, they still contribute significantly to morbidity and hospital admissions. Several factors contribute to the likelihood of birth injuries including macrosomia, cephalopelvic disproportion, dystocia, abnormal presentation, and certain operative deliveries.^1^ Among various types of birth injuries, injuries to the brachial plexus and phrenic nerve can rarely occur.

Perineural cysts, also known as Tarlov cysts, are sac‐like expansions filled with cerebrospinal fluid that develop between the perineurium and endoneurium of nerve roots.^2^ The estimated incidence of perineural cysts is approximately 4.6%, although it may be underestimated.^3^ These cysts are more commonly detected in the sacral region followed by the thoracic, cervical, and lumbar spine.^4^ In most cases, perineural cysts are asymptomatic and are incidentally found during MRIs that are performed for other reasons.^5^ Currently, there are no non‐operative treatments available for managing symptomatic perineural cysts. However, the use of oral or epidural steroid therapy has shown potential in treating such cysts.^6^

In this case report, we describe a neonate with a complex birth injury and an incidental finding of a cervical Tarlov cyst on MRI. The presence of Tarlov cysts in neonates is rare, and their clinical significance is not well understood due to the limited information available. Further studies and case reports may shed more light on this topic.

## 2 CASE PRESENTATION

In this case report, we present the clinical details of a 24‐day‐old female infant who was transferred to our hospital for further evaluation due to suspected diaphragm paralysis.

**FIGURE 1:**
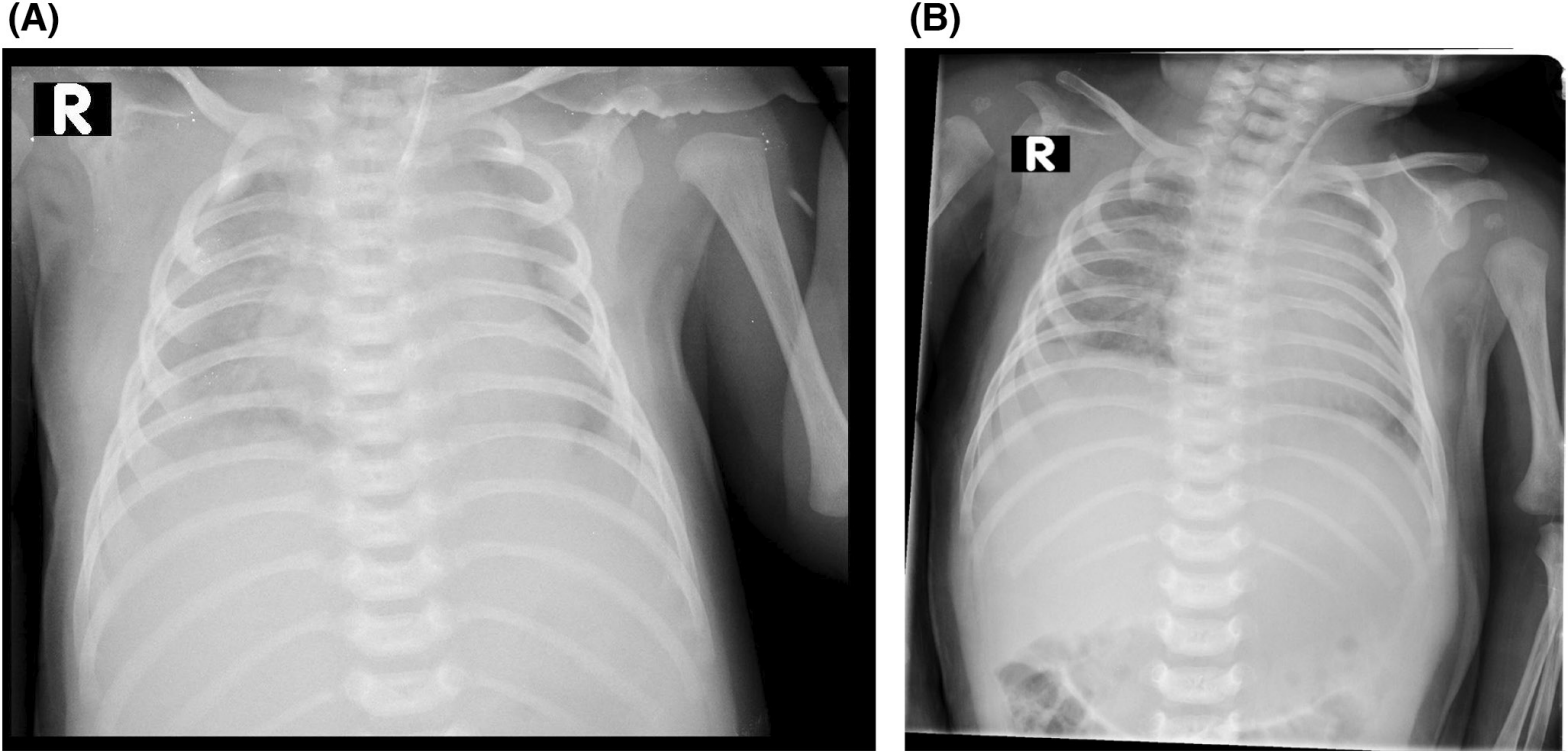
Chest X‐ray (A‐P view) shows the decreased volume of the lung (A, B) and fracture of the posterior of the 6th left ribe due to labor dystocia (white arrow).

**FIGURE 2:**
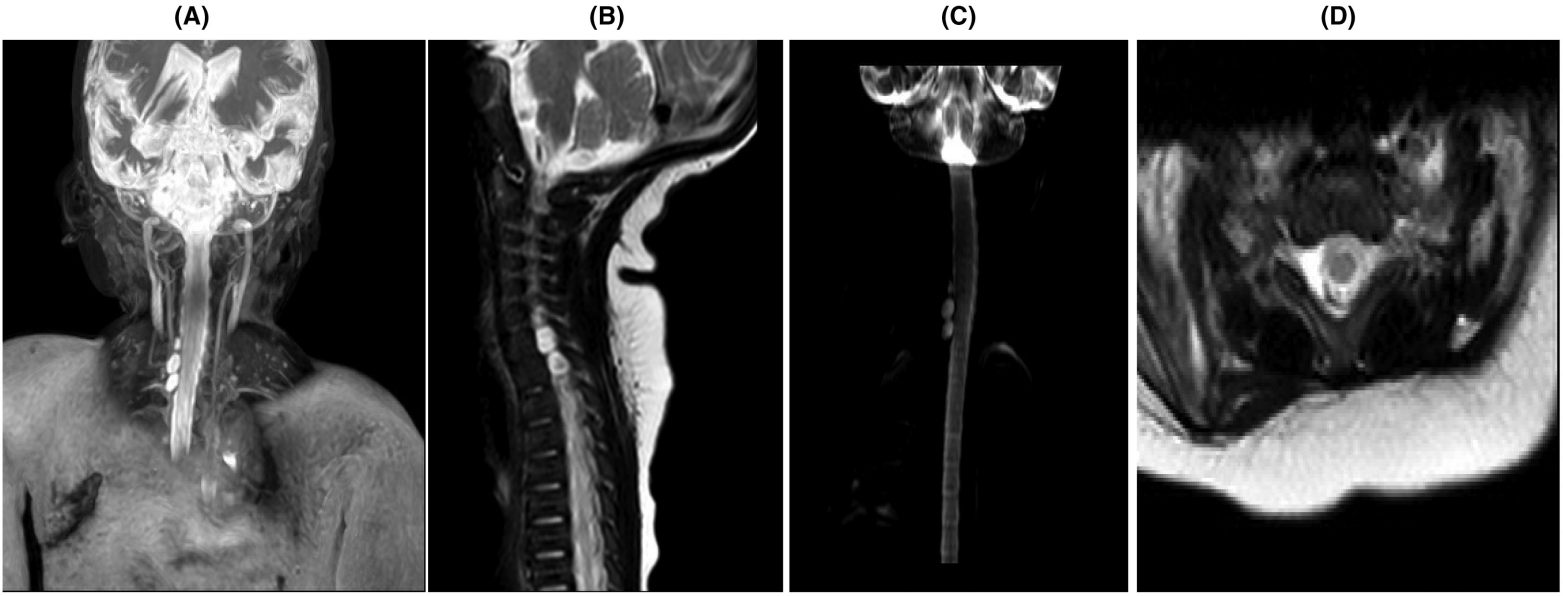
Coronal (A), saggital T2‐weighted (B), myelogram (C), axial T2‐weighted cervical spine (D) show partial injury of C3, C4, C5, C6, and avulsion injury of root of C7, C8, T1 that mimick tarlove cysts.

The infant's mother was a 40‐year‐old with a history of gestational hypothyroidism and poorly controlled gestational diabetes. She had a pre‐pregnancy weight of 85 kg and a body mass index exceeding 30 kg/m^2^. At the end of pregnancy, her weight had increased to 110 kg. The infant was delivered vaginally at 41 weeks of gestational age and was diagnosed with a birth injury due to being large for gestational age, with a birth weight of 5 kg. She also had a low Apgar score at birth. Upon examination at our hospital, the infant was alert with vital signs within normal ranges, including a blood pressure of 85/43 mmHg, pulse rate of 150 beats per minute, respiratory rate of 58 breaths per minute, and a temperature of 36.8°C. She was receiving 8 L per minute of oxygen through a hood and her oxygen saturation was measured at 94%. The sucking reflex and rooting were normal, but the Moro reflex was absent on the right side. The grasping reflex was also absent on the right side. The right upper extremity displayed no active movement, with the elbow held in extension and the forearm pronated. In contrast, the left upper extremity and both lower extremities exhibited normal movement, tone, and reflexes. A chest X‐ray revealed an elevated right diaphragm, indicating diaphragm paralysis and a rib fracture in left side (Figure 1). Sonography further confirmed the elevation of the right diaphragm, absence of diaphragm movement during respiration, and mild pleural effusion on the right side. Additionally, cervical and brain MRIs were performed, revealing a partial injury of C3, C4, C5, and C6 and a complete tear of the right side C7‐C8‐T1 nerve roots mimicking Tarlov cysts (Figure 2). Subsequently, thoracotomy and plication of the right diaphragm were performed as part of the treatment. However, despite being visited by a neurosurgeon, the parents decided against continuing with the treatment process and opted to take the infant home.

## 3 DISCUSSION

Here we describe a neonate who suffered from a complex birth injury, including brachial plexus and phrenic injuries, as well as a rib fracture. Additionally, the neonate was found to have a cervical Tarlov cyst, which may be associated with the birth injury and tear of nerve roots. The patient was diagnosed with macrosomia, which is defined as a birth weight exceeding 4000 g or above the 90th percentile. The mother had gestational diabetes that was not adequately controlled. It is worth noting that approximately 15%–45% of infants born to diabetic mothers experience macrosomia, making it about three times more compared to those of normoglycemic controls.^7^ Maternal age over 35 years old is also considered a risk factor for macrosomia.^8^ Furthermore, pre‐pregnancy overweight, and obesity contribute to the occurrence of macrosomia.^9^ Macrosomia babies are significantly more susceptible to brachial plexus injuries during delivery, with a 20‐fold higher risk compared to babies with normal birth weights.^10^ A significant concern in preventing brachial plexus palsy is the evident link between this condition and macrosomia in diabetic mothers. This connection results in a higher occurrence of complete palsies, leading to more long‐lasting disabilities.^11^ In the literature review, it is reported that 2%–70% of infants with macrosomia are linked to brachial plexus injuries.^8^, ^11^, ^12^ To migrate the risks associated with suspected fetal macrosomia, cesarean delivery may be considered. For women without diabetes, planned cesarean delivery is recommended for suspected fetal macrosomia with estimated weights of at least 5000 g, while for women with diabetes, the threshold is set at 4500 g. However, the decision to perform cesarean delivery for suspected fetal macrosomia remains controversial, as it reduces but does not eliminate the risk of birth trauma and brachial plexus injury.^13^ In our presented case, there were several risk factors for macrosomia, including advanced maternal age, uncontrolled gestational diabetes, and obesity. Considering these factors, it would have been advisable to opt for a cesarian delivery to reduce the potential morbidities and birth trauma.

Neonatal brachial plexus palsy includes Erb's palsy involving upper trunk nerve injury affecting C5 and C6 nerve roots, and Klumpke's palsy, which involves lower trunk nerve injury affecting the C8 and T1 nerve roots. A minority of cases are accompanied by diaphragm paralysis due to phrenic nerve involvement (C3 to C5). The incidence of traumatic brachial plexus palsy is estimated to be around 1.3–1.5 per 1000 live birth.^14^ On the other hand, the incidence of perinatal phrenic nerve palsy is rarer, occurring in approximately 1 in 15,000–30,000 live birth.^15^ In a large cross‐sectional retrospective study from 2004 to 2018, involving 5832 patients diagnosed with brachial plexus palsy resulting from birth trauma, it was observed that 2% of individuals also presented with concurrent diaphragm paralysis.^16^ This indicates that the co‐occurrence of brachial plexus palsy and diaphragmatic paralysis is a relatively rare presentation. Our patient suffers from a complex birth injury involving Erb's palsy, Klumpke's palsy, and diaphragmatic paralysis.

Tarlov cysts are indeed an extremely rare incidental finding in the pediatric population^17^ and there is limited information available especially for this age group. The exact pathogenesis of Tarlov cysts remains unclear. One proposed theory suggests that bleeding into the subarachnoid space leads to the obstruction of venous drainage. This obstruction causes veins to become swollen and eventually rupture, forming the cyst.^18^, ^19^ Trauma has been suggested as a potential risk factor for the Tarlov cyst. Additionally, there may be a genetic susceptibility or predisposition that increases the likelihood of developing a Tarlov cyst.^20^, ^21^ It is important to note that the exact cause of Tarlov cysts is not fully understood. Various theories have been suggested, including ideas related to inflammation, physical injury, congenital factors, the degeneration process, and the possibility of genetic inheritance.^22^ Our patient has experienced birth‐related trauma and inflammation, which may be contributing factors to the development of the Tarlov cyst.

We have reviewed case reports of pediatric patients diagnosed with Tarlov cysts, highlighting their rarity in this age group. Dayyani et al. described an 8‐month‐old infant who presented with irritability and unstable sitting position and was subsequently diagnosed with a Tarlov cyst.^23^ McEvoy et al. reported a 14‐year‐old girl with severe low back pain radiating to the umbilical region and occasional numbness in the right leg was diagnosed with a Tarlov cyst. Conservative treatment failed to yield a response, leading to surgical intervention. During surgery, a cystic fistula involving the root of the nerve was visualized. Following the surgery, all symptoms improved.^24^ Mijalcic et al. presented a case of a 7‐year‐old boy with a history of involuntary urination and occasional back pain radiating to the right leg without a definite diagnosis. Examination revealed neurological abnormalities and MRI confirmed the presence of a large cyst in the spinal canal, along with some form of sacral spina bifida. Surgery was performed with a favorable outcome.^25^ Shams et al. also reported an 11‐year‐old boy, a case of Ehlers‐Danlos syndrome complaining of urine and fecal incontinence. A talov cyst and tethered spinal cord were diagnosed on the lower spine by MRI. Surgical treatment was not successful for him.^26^ Shimauchi‐Ohtaki et al. also reported a 13‐year‐old girl with intractable vomiting and constipation who was diagnosed with a sacral perineural cyst and successfully treated with the operation.^27^ It is important to note that cervical Tarlov cysts are extremely rare and in a retrospective study of 3128 spinal MRIs only 2.2% cervical cyst was found.^4^ Based on our research on PubMed and Google Scholar with the terms “Tarlov cyst,” “perineural cyst,” “pediatric,” “children,” and “cervical” there was no report of a pediatric patient with cervical Tarlov cyst. And there was also no report of a newborn with a Tarlov cyst. So, this is the first case report of a neonate with cervical Tarlov cyst.

## 4 CONCLUSION

The co‐existence of a birth injury and Tarlov cyst adds further complexity to the clinical presentation and management considerations. Given the limited information available on Tarlov cysts in pediatrics, continued research and case reports are necessary to deepen our understanding of the clinical significance, pathogenesis, and optimal management strategies for this unique combination of conditions.

## AUTHOR CONTRIBUTIONS

**Khadijeh sadat Najib:** Conceptualization; writing – original draft; writing – review and editing. **Seyyed Mostajab Razavinejad:** Conceptualization; writing – original draft; writing – review and editing. **Fatemeh Yarmahmoodi:** Conceptualization; writing – original draft; writing – review and editing. **Hamide Barzegar:** Conceptualization; data curation; supervision; writing – original draft; writing – review and editing.

## FUNDING INFORMATION

No funding was obtained for this study.

## CONFLICT OF INTEREST STATEMENT

The authors declare that they have no competing interests.

## ETHICS STATEMENT

The study protocol conformed to the ethical guidelines of the 1975 Helsinki Declaration. The publication of this case was approved by the ethics committee of Shiraz University of Medical Sciences (Approval ID: IR.SUMS.REC.1402.242).

## CONSENT

We have written informed consent obtained from the parents of the patient for publication of this case report.

## Data Availability

All data generated or analyzed during this study are included in this published article.

## References

[1] Brown S , Mozurkewich E . Trauma during pregnancy. Obstet Gynecol Clin North Am. 2013;40(1):47‐57. 23466136 10.1016/j.ogc.2012.11.004

[2] Tarlov IM . Spinal perineurial and meningeal cysts. J Neurol Neurosurg Psychiatry. 1970;33(6):833‐843. 5531903 10.1136/jnnp.33.6.833 PMC493601

[3] Jain M , Sahu NK , Naik S , Bag ND . Symptomatic Tarlov cyst in cervical spine. BMJ Case Rep. 2018;11(1):e228051. 10.1136/bcr-2018-228051 PMC6301451 30567194

[4] Kozłowski P , Kalinowski P , Kozłowska M , et al. Spinal perineural cysts among European patients. J Neurol Surg A Cent Eur Neurosurg. 2021;82(5):463‐467. 33822351 10.1055/s-0040-1722194

[5] Jung KT , Lee HY , Lim KJ . Clinical experience of symptomatic sacral perineural cyst. Korean J Pain. 2012;25(3):191‐194. 22787551 10.3344/kjp.2012.25.3.191 PMC3389325

[6] Mitra R , Kirpalani D , Wedemeyer M . Conservative management of perineural cysts. Spine (Phila Pa 1976). 2008;33(16):E565‐E568. 18628699 10.1097/BRS.0b013e31817e2cc9

[7] Kc K , Shakya S , Zhang H . Gestational diabetes mellitus and macrosomia: a literature review. Ann Nutr Metab. 2015;66(Suppl 2):14‐20. 10.1159/000371628 26045324

[8] Najafian M , Cheraghi M . Occurrence of fetal macrosomia rate and its maternal and neonatal complications: a 5‐year cohort study. ISRN Obstet Gynecol. 2012;2012:353791. 23209925 10.5402/2012/353791 PMC3504382

[9] Wang YW , Chen Y , Zhang YJ . Risk factors combine in a complex manner in assessment for macrosomia. BMC Public Health. 2023;23(1):271. 36750950 10.1186/s12889-023-15195-9 PMC9906846

[10] McFarland LV , Raskin M , Daling JR , Benedetti TJ . Erb/Duchenne's palsy: a consequence of fetal macrosomia and method of delivery. Obstet Gynecol. 1986;68(6):784‐788. 3785790

[11] Van Der Looven R , le Roy L , Tanghe E , et al. Risk factors for neonatal brachial plexus palsy: a systematic review and meta‐analysis. Dev Med Child Neurol. 2020;62(6):673‐683. 31670385 10.1111/dmcn.14381

[12] Dawodu A , Sankaran‐Kutty M , Rajan TV . Risk factors and prognosis for brachial plexus injury and clavicular fracture in neonates: a prospective analysis from The United Arab Emirates. Ann Trop Paediatr. 1997;17(3):195‐200. 9425373 10.1080/02724936.1997.11747886

[13] American College of Obstetricians and Gynecologists Committee on Practice Bulletins—Obstetrics . Practice Bulletin No. 173: fetal Macrosomia. Obstet Gynecol. 2016;128(5):e195‐e209. 27776071 10.1097/AOG.0000000000001767

[14] Chauhan SP , Blackwell SB , Ananth CV . Neonatal brachial plexus palsy: incidence, prevalence, and temporal trends. Semin Perinatol. 2014;38(4):210‐218. 24863027 10.1053/j.semperi.2014.04.007

[15] Stramrood CA , Blok CA , van der Zee DC , Gerards LJ . Neonatal phrenic nerve injury due to traumatic delivery. J Perinat Med. 2009;37(3):293‐296. 19199838 10.1515/JPM.2009.040

[16] Rizeq YK , Many BT , Vacek JC , et al. Diaphragmatic paralysis after phrenic nerve injury in newborns. J Pediatr Surg. 2020;55(2):240‐244. 31757507 10.1016/j.jpedsurg.2019.10.038

[17] Ramadorai UE , Hire JM , DeVine JG . Magnetic resonance imaging of the cervical, thoracic, and lumbar spine in children: spinal incidental findings in pediatric patients. Global Spine J. 2014;4(4):223‐228. 25396102 10.1055/s-0034-1387179 PMC4229374

[18] Sharma M , Velho V , Mally R , Khan SW . Symptomatic lumbosacral perineural cysts: a report of three cases and review of literature. Asian J Neurosurg. 2015;10(3):222‐225. 26396612 10.4103/1793-5482.161177 PMC4553737

[19] Langdown AJ , Grundy JR , Birch NC . The clinical relevance of Tarlov cysts. J Spinal Disord Tech. 2005;18(1):29‐33. 15687849 10.1097/01.bsd.0000133495.78245.71

[20] Paulsen RD , Call GA , Murtagh FR . Prevalence and percutaneous drainage of cysts of the sacral nerve root sheath (Tarlov cysts). AJNR Am J Neuroradiol. 1994;15(2):293‐297. 8192075 PMC8334605

[21] Arora KK , Chaudhary P . Tarlov cyst‐a rare occurrence: a short series of two cases. Int J Res Orthop. 2020;6(3):643.

[22] Marino D , Carluccio MA , di Donato I , et al. Tarlov cysts: clinical evaluation of an italian cohort of patients. Neurol Sci. 2013;34(9):1679‐1682. 23400656 10.1007/s10072-013-1321-0

[23] Dayyani M , Zabihyan S . Giant Tarlov cyst of infancy. World Neurosurg. 2019;123:348‐350. 30576828 10.1016/j.wneu.2018.12.009

[24] McEvoy SD , DiLuna ML , Baird AH , Duncan CC . Symptomatic thoracic Tarlov perineural cyst. Pediatr Neurosurg. 2009;45(4):321‐323. 19713723 10.1159/000235751

[25] Mijalcic M , Djurovic B , Cvrkota I , Jokovic M , Bascarevic V , Micovic M . Tarlov cyst—a rare lesion in children: case report. Childs Nerv Syst. 2019;35:701‐705. 30810854 10.1007/s00381-019-04105-3

[26] Shams S , Issar R , Kadrmas‐Iannuzzi T . A Case of a Tarlov Cyst in a Pediatric Patient With Ehlers‐Danlos Syndrome. Cureus. 2022;14(9):e29009. 36249617 10.7759/cureus.29009 PMC9550184

[27] Shimauchi‐Ohtaki H , Honda F , Nakamura S , Yoshimoto Y . Severe constipation due to sacral perineural cysts in a pediatrics patient: a case report. Surg Neurol Int. 2022;13:317. 35928307 10.25259/SNI_1152_2021 PMC9345110

